# *Madurella tropicana* and *Madurella pseudomycetomatis* identified as new causative agents of black grain eumycetoma in Senegal

**DOI:** 10.1016/j.mmcr.2025.100721

**Published:** 2025-08-06

**Authors:** Maguette Faye, Maodo Ndiaye, Abdou Magib Gaye, Mickey Konings, Wendy Van de Sande, Doudou Sow

**Affiliations:** a Department of Parasitology-Mycology, Faculty of Health Sciences, Gaston Berger University of Saint-Louis, P.O. Box: 234-46024, Saint-Louis, Senegal; b Department of Dermatology, Faculty of Medicine, Cheikh Anta Diop University of Dakar, P.O. Box 5005, Dakar, Senegal; c Department of Pathology, Faculty of Medicine, Cheikh Anta Diop University of Dakar, P.O. Box 5005, Senegal; d Erasmus MC, University Medical Center Rotterdam, Department of Medical Microbiology and Infectious Diseases, Dr. Molewaterplein 40, 3015GD, Rotterdam, the Netherlands

**Keywords:** Eumycetoma, Black grain, *Madurella tropicana*, *Madurella pseudomycetomatis*, Senegal

## Abstract

This study reports *Madurella tropicana* and *Madurella pseudomycetomatis* as pathogens causing eumycetoma in Senegal. The dominant species, *Madurella mycetomatis*, shares morphological features with both. Accurate species-level identification is crucial for treatment and epidemiology. Molecular diagnostic tools, especially ITS gene sequencing, enabled the identification of these species. Itraconazole treatment showed a favorable outcome in a patient infected with *Madurella pseudomycetomatis*, leading to sinus closure.

## 1 Introduction

Mycetoma was recognized as a Neglected Tropical Disease by the World Health Organisation in 2016. It is characterized by large tumorous lesions in the subcutaneous tissues, especially in the lower limbs, resulting in severe disabilities [1]. Characteristic of this disease is that the causative agents reside in granules in the tissue called grains [2,3]. Mycetoma can be caused by bacteria (actinomycetoma) and fungi (eumycetoma) and there are more than 90 different causative agents implied. Regional differences in the etiology are noted, but for most causative agents it is not completely known in which regions they cause mycetoma. In Latin-America, actinomycetoma is more common than eumycetoma. In Africa, the most endemic areas for eumycetomas are Senegal and Sudan [4,5]. *Madurella mycetomatis* is the main species found in Sudan [6]. While in Senegal, *Madurella mycetomatis*, *Falciformispora senegalensis*, and several other agents have been identified as causative organisms of eumycetomas based on culture results [[7], [8], [9]]. However, in other endemic region it was already demonstrated that a more diverse spectrum of causative agents is identified when molecular tools are used for the identification of causative agents [1,10]. This study reports two cases of mycetoma caused by *Madurella tropicana* and *Madurella pseudomycetomatis* isolated and identified for the first time in Senegal.

## 2 Case presentation

**Fig. 1:**
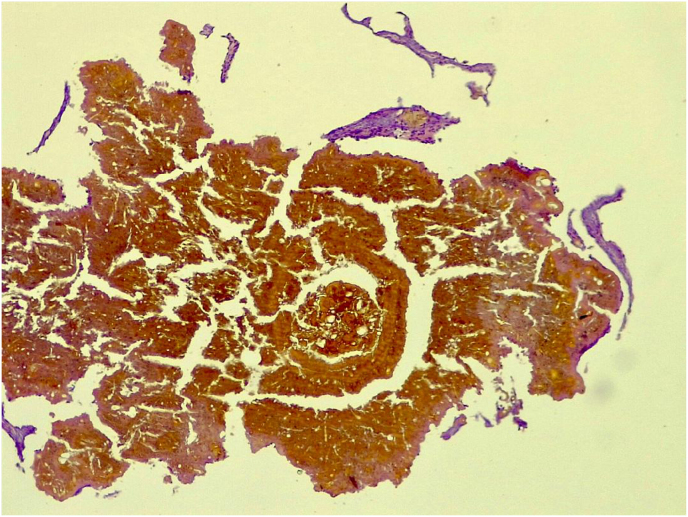
Grain of *Madurella tropicana* in hematoxylin and eosin stain were initially identified as *Madurella mycetomatis*.

**1st case:** A 41-year-old, male, farmer from Podor, located in the Saint Louis region of Senegal and currently residing in Rufisque (Dakar), presented with a history of a thorn prick injury to the left leg that occurred on June 15, 2001. Twenty years later, he developed inflammatory swelling associated with multifistulized nodular lesions measuring over 10 cm in diameter, from which black grains were discharged. The patient's past medical and surgical history was unremarkable. Two punch biopsies were obtained using a 5 mm punch under local anesthesia. One specimen was fixed in formalin for histopathological analysis, while the other was placed in saline for mycological and molecular investigations. Histological sections were prepared and stained with hematoxylin and eosin (H&E), revealing diffuse, cement-like, rounded, and multilobulated brown grains-features characteristic of *Madurella mycetomatis* (Fig. 1). Radiographic imaging demonstrated osseous involvement. Direct microscopic examination revealed black, hard grains containing vesicle-like structures. The grains were inoculated on Sabouraud dextrose agar supplemented with chloramphenicol (SC); however, after 12 weeks of incubation, no fungal growth was observed. Subsequent molecular identification was performed by extracting DNA directly from the grains using the ZR Fungal/Bacterial DNA MiniPrep Kit. The internal transcribed spacer (ITS) region was amplified using primers V9G (5′-TTACGTCCCTGCCCTTTGTA-3′) and LS266 (5′-GCATTCCCAAACAACTCGACTC-3′). PCR products were sequenced using the BigDye Terminator v3.1 Ready Reaction Cycle Sequencing Kit (Applied Biosystems, Waltham, MA, USA), following the manufacturer's protocol. Sequence analysis showed 99.55 % identity (223/224 bp) to the ITS region of the holotype strain *Madurella tropicana* CBS201.38 (GenBank accession number MK926824.1). The patient was initiated on itraconazole therapy at the dose of 400 mg per day; however, he was lost to follow-up after four months of treatment.

**Fig. 2:**
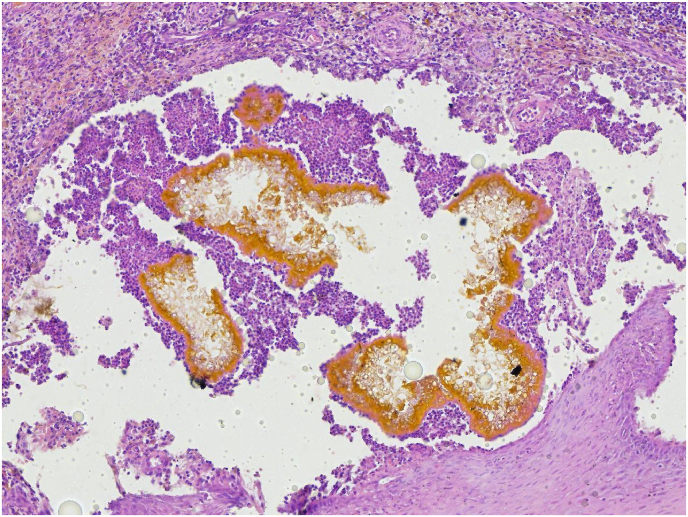
Grain of *Madurella pseudomycetomatis* in hematoxylin and eosin stain were initially identified as *Leptosphaeria senegalensis*.

**Fig. 3:**
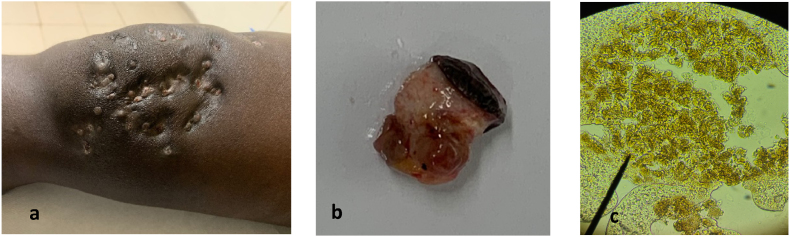
a. Clinical presentation; b. Mass sectioned with black grains inside the tissues; c. Microscopic view of grains (X40).

**Fig. 4:**
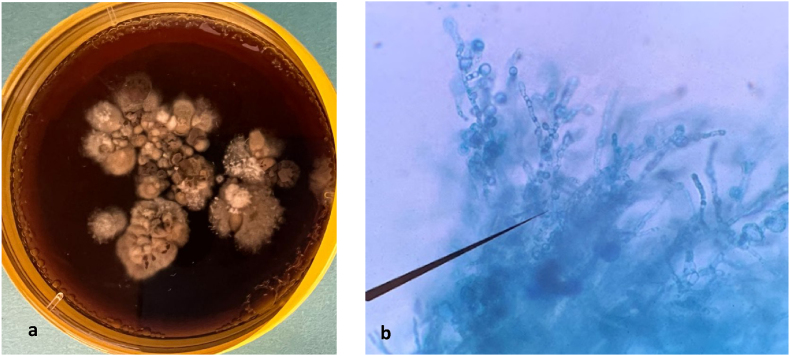
a. *Madurella pseudomycetomatis* colony on SC at 37 °C 6 days; b. Microscopic culture.

**2nd case:** A 24-year-old, male, truck transporter from Kebemer, in the Louga region of Senegal, with no prior medical history and no recollection of previous trauma, presented with a chronic inflammatory swelling featuring multiple fistulas. The lesion, measuring over 10 cm in diameter, had progressed over a 10-year period and was associated with the discharge of black grains (Fig. 3a and b). Initially, the patient attempted self-treatment with traditional medicine, including oral and topical powdered. Ultrasound imaging of the knee revealed multiple superficial fluid-filled cavities containing echogenic grains, surrounded by a hypervascular rim with granulomatous features suggestive of a fungal mycetoma. A right inguinal lymph node enlargement measuring 16 × 8 mm was also noted. A punch biopsy was performed using 8 mm punch. Tissue samples were divided, with one part fixed in formalin for histopathological analysis and the other placed in saline for culture and molecular investigations. Direct microscopic examination of grains in a 20 % potassium hydroxide (KOH) solution revealed soft black grains (Fig. 3c). Histopathological examination revealed a marked polymorphous inflammatory reaction in the dermis, characterized by a massive infiltration of neutrophils forming micro-abscesses centered around numerous fungal-like grains. These findings were consistent with an eumycetoma caused by *Leptosphaeria senegalensis* Fig. 2. After five days of incubation at 37 °C on Sabouraud dextrose agar supplemented chloramphenicol, a beige to ochre-brown colony was observed. The colony had a velvety, soft texture with an irregular, folded margin and produced a brownish pigment (Fig. 4a). Microscopic examination showed thick, septate hyphae with terminal and intercalary subglobose chlamydospores, which are characteristic of *Madurella. sp.* (Fig. 4b). Genomic DNA was extracted from the fungal isolate, and the internal transcribed spacer (ITS) region was amplified using primers V9G and LS266. Sequencing revealed 100 % identity with *Madurella pseudomycetomatis* strain CBS129177 (GenBank accession number MK926821.1; 436/436 nt match). The patient was treated with oral itraconazole at a dosage of 400 mg/day for six months. The treatment was well tolerated, with no reported side effects. Clinical improvement was observed, including closure of the draining fistulas. However, due to financial constraints, the patient was unable to undergo the planned surgical intervention and was subsequently lost to follow-up.

## 3 Discussion

Research on mycetoma pathogens has intensified in recent years [11,12]. In Senegal, among black-grain eumycetomas, *Madurella mycetomatis* is the predominant species identified by culture-based methods, accounting for approximately 52 % of cases [6,7]. However, *Madurella mycetomatis* is not the sole causative agent within the *Madurella* genus capable of inducing mycetoma. Its sibling species *Madurella pseudomycetomatis*, *Madurella tropicana*, and *Madurella fahalii* are morphologically indistinguishable from *Madurella mycetomatis* [4].

In this study, molecular identification techniques were used to accurately determine the responsible fungal species. Our results show that *Madurella pseudomycetomatis* and *Madurella tropicana* are also endemic in Senegal. However, in the case of *Madurella pseudomycetomatis*, histopathological examination initially suggested a diagnosis of eumycetoma caused by *Leptosphaeria senegalensis*. Molecular analysis based on ITS region sequencing of the isolated strain later confirmed the pathogen as *Madurella pseudomycetomatis*. Similarly, *Madurella tropicana* had previously been misidentified as *Madurella mycetomatis* due to their similar histopathological features. Morphologically, *Madurella tropicana* is indistinguishable from *Madurella mycetomatis*, and the only known difference lies in their optimal growth temperatures: *Madurella tropicana* grows best at 30 °C, whereas *Madurella mycetomatis* and *Madurella pseudomycetomatis* grow optimally at 37 °C. Moreover, no differences in antifungal susceptibility profiles have been reported among these species [4]. This discrepancy between histopathological and molecular findings highlights the limitations of morphological methods, which—despite their usefulness—can lead to misidentifications due to similar granulomatous characteristics shared by several fungal species causing eumycetoma. It also emphasizes the importance of incorporating molecular biology tools for accurate species-level identification, especially in cases involving lesser-known species such as *Madurella pseudomycetomatis* or *Madurella tropicana*, which are prone to misdiagnosis [3,5].

In our study, only *Madurella pseudomycetomatis* was successfully isolated after only five days of grain incubation on a Sabouraud chloramphenicol (SC) agar at 37 °C. This is much faster than the two weeks of culture required on a dextrose agar (SDA) of Sabouraud at 37 °C, based on data from Nyuykonge [13]. Obtaining a positive culture is important in the study of mycetoma for a more reliable identification of the causative pathogens. Indeed, molecular analysis performed on isolated fungal strains provides more accurate results than analysis performed directly on grains collected from the patient [14].

Patients in this study were treated with oral itraconazole at a dose of 400 mg/day. The patient infected with *Madurella tropicana* disappeared after four months of treatment. The patient with *Madurella pseudomycetomatis* responded well to therapy, with closure of fistula pathways within the first six months of treatment. However, due to financial constraints, the patient could not undergo the planned surgery.

Accurate species identification is crucial, to gain more insight in the epidemiology and treatment response of the different eumycetoma causative agents. The findings reported here, demonstrate that Senegal is one of the countries where *Madurella pseudomycetomatis* and *Madurella tropicana* are endemic and demonstrate that at least the patient with mycetoma caused by *Madurella pseudomycetomatis* responded in a similar way to itraconazole treatment as reported for patients infected with *Madurella mycetomatis.* With these two reports, the presence of *Madurella pseudomycetomatis* and *Madurella tropicana* should also be considered as a possible etiology of eumycetoma in Senegal.

## CRediT authorship contribution statement

**Maguette Faye:** Writing – review & editing, Methodology, Investigation, Formal analysis. **Maodo Ndiaye:** Writing – review & editing, Investigation, Conceptualization. **Abdou Magib Gaye:** Writing – review & editing, Investigation. **Mickey Konings:** Writing – review & editing, Investigation. **Wendy Van de Sande:** Writing – review & editing, Project administration, Investigation. **Doudou Sow:** Supervision, Project administration, Methodology, Investigation, Conceptualization.

## Ethical form

This study has been approved by the National Committee of Ethics for Health Research (CNERS). The anonymity and confidentiality of the data collected will be respected.

## Declaration of generative AI and AI-assisted technologies in the writing process

During the preparation of this manuscript, the authors used ChatGPT to assist with the English translation of the text in a form suitable for a scientific article. Following the use of this tool/service, the authors reviewed and edited the content as needed and take full responsibility for the final version of the publication.

Funding

These cases were identified within our Dioraphte sponsored project “developing a novel point-of-care test for mycetoma”.

## Declaration of competing interest

No competing interests to declare.
